# Case report: Disseminated herpes simplex virus 1 infection and hemophagocytic lymphohistiocytosis after immunomodulatory therapy in a patient with coronavirus disease 2019

**DOI:** 10.3389/fmed.2022.1053012

**Published:** 2022-11-21

**Authors:** Elvio Mazzotta, Juan Fiorda Diaz, Marco Echeverria-Villalobos, Gregory Eisinger, Sarah Sprauer, Arindam Singha, Michael R. Lyaker

**Affiliations:** ^1^Department of Anesthesiology, The Ohio State University Wexner Medical Center, Columbus, OH, United States; ^2^Division of Pulmonary, Critical Care, and Sleep Medicine, Department of Internal Medicine and Emergency Medicine, The Ohio State University Wexner Medical Center, Columbus, OH, United States

**Keywords:** human herpes simplex virus 1, COVID-19, liver failure, hemophagocytic lymphohistiocytosis, tofacitinib

## Abstract

Corticosteroids and immunomodulatory therapies are widely used to treat patients with severe coronavirus disease 2019 (COVID-19). Janus kinase (JAK) inhibitors such as tofacitinib have been recently studied as adjuvants in the treatment of COVID-19. Although immunomodulatory therapies may be linked to decreased mortality rates in the acute phase, subsequent severe infectious complications may result from them. We describe a case of a multiorgan system failure secondary to disseminated primary herpes simplex virus 1 (HSV-1) infection and hemophagocytic lymphohistiocytosis (HLH) following treatment with tofacitinib and high-dose dexamethasone therapy for severe COVID-19. Early diagnosis and treatment of these life-threatening conditions may have a significant impact on COVID-19 patients’ outcomes.

## Introduction

The hallmark of severe coronavirus disease 2019 (COVID-19) is an aggressive pro-inflammatory response known as “cytokine storm” (1). Studies have shown that immune system dysregulation and hyperinflammation are associated with increased mortality in severely ill patients (1, 2). Multiple studies suggest that glucocorticoids, Janus kinase (JAK) inhibitors, and interleukin 6 (IL-6) inhibitors may be associated with decreased mortality in critically ill COVID-19 patients (3, 4). However, the immunosuppressive state induced by these agents may increase the risk of secondary infections (5, 6). Herpes simplex virus (HSV) reactivation is a widely known complication in critically ill patients in the intensive care unit (ICU) (7). Nevertheless, disseminated HSV infection and subsequent potentially lethal complications such as hemophagocytic lymphohistiocytosis (HLH) are very uncommon (8, 9).

Hemophagocytic lymphohistiocytosis is a rare and life-threatening condition characterized by an uncontrolled hyper-inflammatory response with hyperactivation of histiocytes leading to hemophagocytosis. Diagnostic criteria include fever, organomegaly, cytopenia, hypertriglyceridemia, and extremely high ferritin (10). Risk factors include, but are not limited to, genetic mutations, immunodeficiency, infections, and malignancy (11). Published literature on HLH treatment and outcomes is limited considering its low incidence. We report a case of disseminated HSV-1 associated with HLH in a patient recently treated with steroids and tofacitinib for COVID 19.

## Case presentation

A 58-year-old Caucasian female without significant past medical history presented to an outside emergency department with a complaint of abdominal pain. She had been recently admitted with respiratory failure secondary to COVID-19 pneumonia. During her hospital course, she was treated with 15 L/min of oxygen by high flow nasal cannula and tofacitinib (10 mg twice daily for 10 days), remdesivir (200 mg on day 1 follow by 100 mg for 5 days), and dexamethasone (6 mg/daily). After 10 days of a relatively uneventful admission, she was discharged home on 3 L of oxygen.

Five days after discharge, the patient presented to an outside hospital emergency department with severe abdominal pain that steadily worsened. Initial laboratory results (Table 1) were notable for pancytopenia, severe acute liver injury, and elevation of inflammatory makers including lactate dehydrogenase (LDH) and ferritin. Her hepatitis panel (including serology for hepatitis A, B, C, cytomegalovirus, and Epstein-Barr virus), and reverse transcriptase polymerase chain reaction (RT-PCR) for severe acute respiratory syndrome coronavirus 2 (SARS-CoV-2) were negative. Computerized tomography (CT) of abdomen and pelvis showed hepatic steatosis without others abdominopelvic findings. Alcohol abuse and acetaminophen ingestion were ruled out. Consequently, the patient was admitted with acute liver injury of unknown origin and concomitant oliguric acute kidney injury (AKI) that subsequently resulted in transfer to our institution for higher level of care.

**TABLE 1:** Initial laboratory results.

Laboratory test (normal ranges, units)	Results
WBC count (3.99–11.19 K/μL)	3.00
RBC count (3.91–5.04 M/μL)	3.76
Hemoglobin (11.4–15.2 g/dL)	11.1
Hematocrit (34.9–44.3%)	33.2
Platelet count (150–393 K/μL)	42
PT (11.9–14.2 s)	32.1
INR (0.9–1.1)	3.2
PTT (24.0–34.3 s)	66.2
Creatinine (0.50–1.20 mg/dL)	5.45
Bilirubin direct (<0.3 mg/dL)	1.2
Bilirubin total (<1.5 mg/dL)	2.1
ALP (32–126 U/L)	181
ALT (9–48 U/L)	7,750
AST (10–39 U/L)	13,680
Ferritin (10.0–291.0 ng/mL)	98,821
LDH total (100–190 U/L)	16,190
Triglycerides (<150 mg/dL)	126

WBC, white blood cells; RBC, red blood cells; PT, prothrombin time; INR, international normalized ratio; PTT, partial thromboplastin time; ALP, alkaline phosphatase; ALT, alanine transaminase; AST, aspartate transaminase; LDH, lactate dehydrogenase.

**FIGURE 1:**
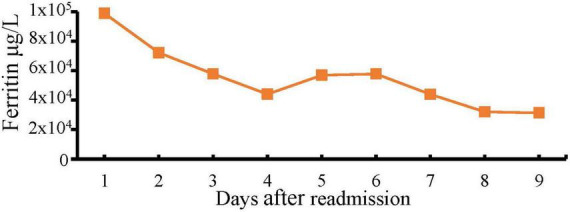
Down-trending ferritin levels after hospital admission.

Shortly after transfer to our institution, the patient developed a rapidly progressive encephalopathy with respiratory distress and septic shock requiring ICU admission, mechanical ventilation, and hemodynamic support. Vancomycin resistant enterococcus (VRE) bacteremia was diagnosed, and the patient was started on daptomycin (10 mg/kg/daily). HLH was initially suspected due to the extremely high ferritin levels. However, a moderate predictive score with normal triglycerides and down-trending ferritin levels delayed the definitive diagnosis (Figure 1) (12).

**FIGURE 2:**
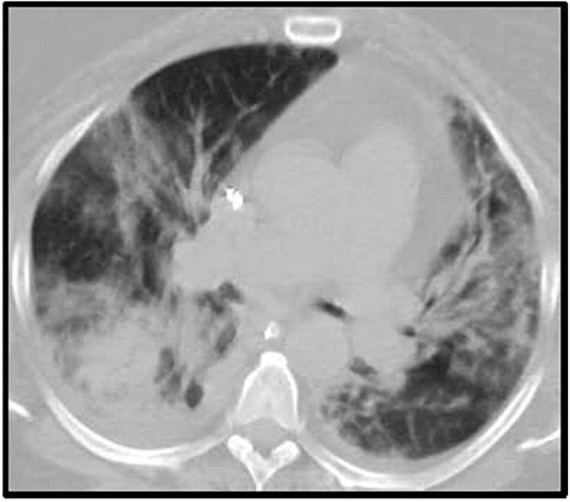
Chest CT after ICU admission. Description: Bilateral multifocal irregular patchy and confluent ground glass and consolidative opacities with associated predominantly bilateral lower lobe irregular reticulations. Small **(right)** and trace **(left)** pleural effusion. CT, computerized tomography; ICU, intensive care unit.

Furthermore, a rash involving her inguinal area was noted. Both, lesion swab and serum PCR, were positive for herpes simplex virus 1 (HSV-1). Primary infection was confirmed with a positive HSV-1 I gM and negative IgG. In addition, chest CT showed bilateral multifocal pneumonia, that had progressed from her prior imaging during COVID admission (Figure 2). Bronchoscopy was performed and notable for diffusely erythematous friable bronchial mucosa. Cytopathology of the bronchial lavage fluid was notable for multinucleated cells with glassy chromatin suggestive of HSV-1 viral pneumonitis (13).

Central nervous system (CNS) involvement of HSV-1 infection was strongly suspected, but lumbar puncture was deferred due to worsening coagulopathy. However, the patient was empirically treated with meningitis dosing of acyclovir (10 mg/kg twice daily).

On admission day 7, increased triglyceride levels and an updated H-score (used for diagnosis of HLH) indicated more than 99% chances of HLH this time (fever 101.1-102.9, Pancytopenia 3 lineages, ferritin >6,000 ng/mL, triglyceride 500 mg/dL, fibrinogen 110 mg/dL, AST 5,470 U/L) (14).

Bone marrow biopsy was not performed due to clinical instability, coagulopathy, and very high pretest probability for HLH. In consultation with hematology, empiric immunosuppressive therapy with intravenous dexamethasone (40 mg/day) was started.

**FIGURE 3:**
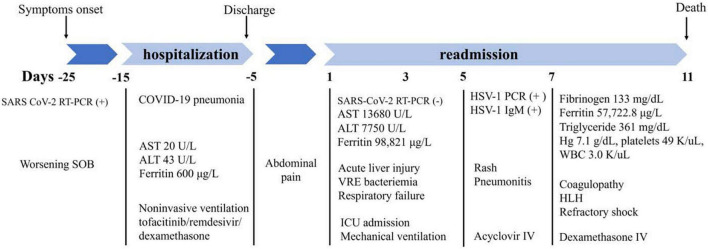
Flow chart of significant clinical events and outcomes. SARS, severe acute respiratory syndrome; COVID-19, coronavirus disease 2019; RT-PCR, reverse transcriptase polymerase chain reaction; SOB, shortness of breath; AST, aspartate transaminase; ALT, alanine transaminase; VRE, vancomycin resistant enterococcus; ICU, intensive care unit; HSV-1, herpes simplex virus 1; IV, intravenous; Hg, hemoglobin; WBC, white blood cells; HLH, hemophagocytic lymphohistiocytosis.

On admission day 10, the patient’s clinical condition continued to deteriorate with worsening shock, AKI requiring continuous renal replacement therapy (CRRT), refractory metabolic acidosis, escalating ventilator requirements, and loss of brain stem reflexes. Head CT showed a trace of right frontal convexity subarachnoid hemorrhage and Abdomen/Pelvis CT displayed iliopsoas hematoma. Given the poor prognosis, the family made the decision to transition to comfort measures and on hospital day 11, patient expired (Figure 3).

## Discussion

We described a case of a disseminated primary HSV-1 infection complicated by HLH in a patient treated with tofacitinib and dexamethasone for COVID-19. While treatment with corticosteroids is supported by high quality evidence (4) and has become standard of care for patient with respiratory failure secondary to COVID-19, the role of other adjunctive immunomodulatory agents in patients with a hyperinflammatory profile remains controversial (12). Of concern, reduced immune response induced by immunomodulators may be associated with an increased risk of secondary bacterial, viral, and fungal infections in COVID-19 patients (6).

Our patient was treated with tofacitinib, a specific JAK inhibitor widely used for treatment of rheumatoid arthritis and a common alternative to tocilizumab and baricitinib. In a randomized clinical trial including 289 patients admitted with COVID-19 pneumonia, tofacitinib significantly reduced the cumulative incidence of death or respiratory failure at 28 days when compared with placebo (RR 0.63; 95% confidence interval [CI], 0.41 to 0.97; *p* = 0.04) (15). The risk of severe infections was not greater in the tofacitinib group in comparison with placebo; however, a significant increase in transaminase levels was reported in the tofacitinib group (15). Moreover, suppressed lymphocyte activation and proliferation induced by this drug may result in a higher susceptibility to HSV-1 infection (16–19). The fulminant disease course may be entirely or partly due to use of JAK inhibitor and its risk of severe secondary infection. However, the patient also had several major risk factors for severe disease and poor outcome of COVID-19.

Herpes simplex virus reactivation is a well-known complication in critically ill patients in the ICU; however, disseminated HSV-1 infection with concomitant hepatitis and pneumonitis is an uncommon clinical entity linked to high mortality rates (8). Fatal cases of disseminated HSV-1 infection have been reported elsewhere in cases of COVID-19 infection. Busani et al. recently described acute liver failure and HSV-1 infection in two male patients who received tocilizumab and corticosteroids as part of their therapy for COVID-19 (5). The authors reported refractory metabolic acidosis, irreversible shock, and impaired liver function as the main causes of death in both patients. Both patients received similar therapies including hydroxychloroquine, azithromycin, tocilizumab, and methylprednisolone. Recognition of disseminated HSV-1 was delayed in both patients, with diagnosis on days 15 and 33 of admission, respectively (5).

To confirm the diagnosis of disseminated HSV, invasive procedures such as liver biopsy and spinal puncture can be considered. However, these procedures carry an increased risk of bleeding. Therefore, it is crucial to weight risks and benefits in a coagulopathic patient.

In adults, the most common triggers for HLH are infection or alteration in immune homeostasis like autoimmune diseases and cancer (10). Primary viral infections or reactivation have been commonly identified as the main causes of HLH, being Epstein-Barr virus the most commonly reported followed by herpes simplex virus and cytomegalovirus (10, 20). Growing evidence suggests an increased incidence of HLH in patients with severe COVID-19. However, most of these cases are clinically diagnosed and lack confirmatory testing (i.e., bone marrow biopsy) due to patients’ instability (21). The cornerstone of HLH management includes the treatment of the triggering condition, aggressive immunosuppression, and corticosteroids (10, 22). In our patient, the course of events of the COVID-19 and HLH diagnoses did not overlap, making the disseminated primary HSV its most likely trigger. Extremely high ferritin level with pancytopenia raised primary concerns for secondary HLH. However, down-trending ferritin could have delayed this diagnosis. Additionally, the risks and benefits of instituting aggressive immunosuppression for the treatment of HLH were challenging to weigh in a patient with disseminated HSV-1 and enterococcal bacteremia. It is unknown whether earlier steroid treatment would have improved the outcome or worsened the infectious processes.

## Conclusion

To the best of our knowledge, our case report may be the first case reporting a patient with a disseminated HSV-1 infection associated with HLH after treatment for COVID-19. Immunomodulatory therapies for moderate-to-severe COVID-19 may place patients at higher risk of severe secondary infections. Disseminated HSV-1 and acute liver failure are associated with high mortality rates. Future research on immunomodulatory therapies for COVID-19 management may elucidate the associated risks and benefits in patients with potential predisposing factors for severe systemic diseases.

## Data availability statement

The original contributions presented in this study are included in the article/supplementary material, further inquiries can be directed to the corresponding author.

## Ethics statement

Ethical review and approval was not required for the study on human participants in accordance with the local legislation and institutional requirements. The patients/participants provided their written informed consent to participate in this study. Written informed consent was obtained from the individual(s) for the publication of any potentially identifiable images or data included in this article.

## Author contributions

All authors listed have made a substantial, direct, and intellectual contribution to the work, and approved it for publication.

## Abbreviations

COVID-19: coronavirus disease 2019
HSV: herpes simplex virus
ICU: intensive care unit
HLH: hemophagocytic lymphohistiocytosis
LDH: lactate dehydrogenase
PCR: polymerase chain reaction
RT- PCR: reverse transcriptase polymerase chain reaction
SARS-CoV-2: severe acute respiratory syndrome coronavirus 2
CT: computerized tomography
AKI: acute kidney injury
VRE: Vancomycin resistant enterococcus
BID: twice daily
CRRT: continuous renal replacement therapy.
